# A Canonical Laminar Neocortical Circuit Whose Bottom-Up, Horizontal, and Top-Down Pathways Control Attention, Learning, and Prediction

**DOI:** 10.3389/fnsys.2021.650263

**Published:** 2021-04-23

**Authors:** Stephen Grossberg

**Affiliations:** Graduate Program in Cognitive and Neural Systems, Departments of Mathematics and Statistics, Psychological and Brain Sciences, and Biomedical Engineering, Center for Adaptive Systems, Boston University, Boston, MA, United States

**Keywords:** neocortex, attention, perceptual grouping, category learning, 3D vision, working memory, catastrophic forgetting, adaptive resonance theory

## Abstract

All perceptual and cognitive circuits in the human cerebral cortex are organized into layers. Specializations of a canonical laminar network of bottom-up, horizontal, and top-down pathways carry out multiple kinds of biological intelligence across different neocortical areas. This article describes what this canonical network is and notes that it can support processes as different as 3D vision and figure-ground perception; attentive category learning and decision-making; speech perception; and cognitive working memory (WM), planning, and prediction. These processes take place within and between multiple parallel cortical streams that obey computationally complementary laws. The interstream interactions that are needed to overcome these complementary deficiencies mix cell properties so thoroughly that some authors have noted the difficulty of determining what exactly constitutes a cortical stream and the differences between streams. The models summarized herein explain how these complementary properties arise, and how their interstream interactions overcome their computational deficiencies to support effective goal-oriented behaviors.

## 1. Introduction: Why Does The Cerebral Cortex Have Layers?

This article discusses a particular combination of feedforward and feedback pathways in the cerebral cortex that plays a crucial role in combining information to make adaptive decisions and predictions. In so doing, it falls directly within the purview of the Frontiers Research Topic on *Feedforward and Feedback Processing in the Cerebral Cortex: Connectivity and Function* to which it contributes. This circuit design benefits from the fact that all perceptual and cognitive neocortical circuits are organized into *layers*, typically six of them (Brodmann, 1909; Martin, 1989). These layers enable a canonical neocortical circuit to be realized whose specializations carry out multiple functions of biological intelligence.

Given the diversity of functions carried out by specialized neocortical circuits, it is of interest to ask what processes are shared across variations in this canonical circuit. Grossberg (1999) identified several general processes that all laminar neocortical circuits seem to share. These include:

(1) the bottom-up adaptive filtering process whereby neocortex compresses, or categorizes, familiar information in order to recognize it;
(2) the horizontal grouping process whereby neocortex binds distributed features into coherent object representations that are sensitive to the contrasts and spatial arrangement of their inducing features;
(3) the top-down matching process whereby neocortex uses learned expectations to focus attention upon critical feature patterns that predict valued outcomes, while suppressing irrelevant features; and
(4) the developmental and learning processes whereby neocortex shapes its circuits in response to environmental constraints, while dynamically stabilizing adaptations, such as those in items (1)–(3), to avoid catastrophic forgetting.

When these feedforward and feedback processes are combined into a canonical laminar cortical circuit, they clarify how seemingly unrelated psychological properties are mechanistically related. For example, they clarify how the fact that neocortex can develop and learn in a self-stabilizing way implies why top-down attention requires matched bottom-up inputs or volitional signals to fully activate cortical cells, whereas horizontal groupings do not.

This article provides an overview and unified analysis of laminar cortical models of multiple types of biological intelligence, including 3D vision and figure-ground perception; attentive category learning and decision-making; speech perception; and cognitive working memory (WM), planning, and prediction. These models are consistent with descriptions and statistical analyses of cortical connectivity patterns (e.g., Gilbert and Wiesel, 1979; Felleman and Van Essen, 1991; Markov et al., 2013; Vezoli et al., 2021), as well as recent data about “visual evoked responses in laminar recordings from six cortical areas in awake mice” (Barzegaran and Plomp, 2021).

The current models additionally explain how neurophysiological dynamics in specific feedforward and feedback anatomies generate emergent properties that enable quantitative properties of behavior to be explained and simulated on the computer. Moreover, although the anatomies in these models are all variations of known canonical cortical circuits, the explanations herein of how their variations realize psychological processes as distinct as 3D vision and figure-ground perception; attentive category learning and decision making; speech perception; and cognitive working memory, planning, and prediction, cannot be explained using purely anatomical analyses, and stand as testable predictions for further experimentation.

## 2. Perceptual Groupings Form Preattentively and Automatically

### 2.1. Boundary and Surface Processes Are Computationally Complementary

The visual cortex is designed to enable us to consciously see objects and scenes in the world. Objects percepts are not just jumbles, or “bags,” of features. Rather, spatially distributed features are bound together into emergent structures within an object percept. The two most important emergent structures during visual perception of a depthful world are boundary groupings and surface representations (Grossberg, 1994, 1997; Grossberg and Pessoa, 1998; Grossberg et al., 2002; Grossberg and Hong, 2006; Grossberg and Huang, 2009).

**Figure 1:**
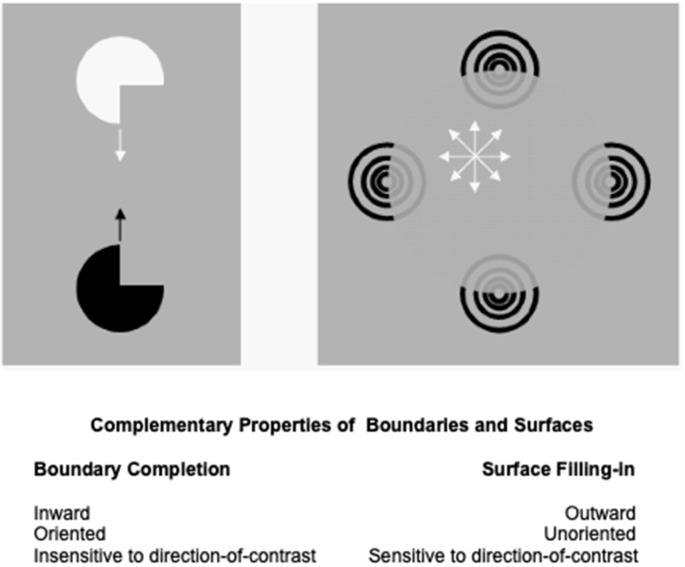
Boundary completion and surface filling-in obey computationally complementary laws. See the text for details (Reprinted with permission fromGrossberg, 2021).

**Figure 2:**
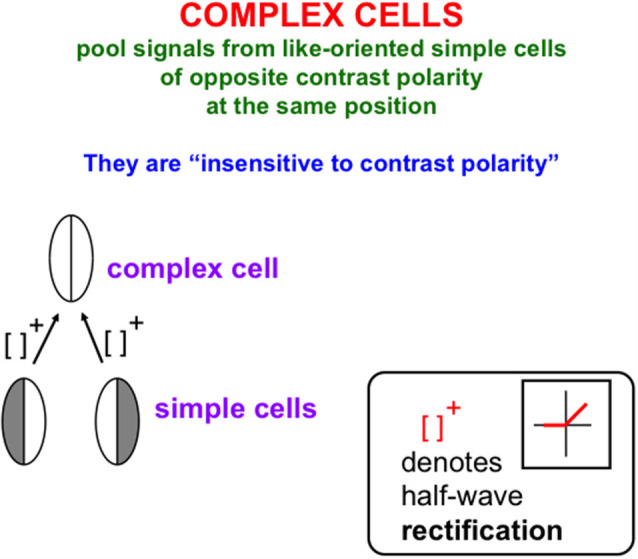
Oppositely-polarized simple cells at the same position with similar orientational preferences input to a complex cell that responds to both contrast polarities, after their activities are thresholded, or half-wave rectified, as they generate output signals (Reprinted with permission from Grossberg, 2021).

The processes that complete boundaries and fill-in surfaces obey computationally *complementary* laws. As summarized in Figure 1, boundary formation occurs *inward* between pairs or greater numbers of inducers in an *oriented* fashion. Boundaries are *insensitive* to direction-of-contrast, or contrast polarity, in the sense that they can group inducers with opposite contrasts relative to an inducing scenic background. In Figure 1, boundaries pool opposite directions-of-contrast at different positions. More generally, boundaries pool opposite directions-of-contrast at *every* position, starting in cortical area V1 at complex cells that sum up opposite polarity signals from pairs of like-oriented simple cells at that position (Figure 2). Because boundaries pool signals from opposite contrast polarities, they cannot tell the difference between light and dark. As a result, “all boundaries are invisible” (Grossberg, 1994, 1997).

In contrast, the process that fills in surface brightness and color spreads *outward* from individual inducers in an *unoriented* manner until a boundary blocks its further spread. Filling-in is also *sensitive* to direction-of-contrast, or contrast polarity, because filled-in brightnesses and colors may be consciously seen (Figure 1). Thus, all conscious percepts of visible qualia are surface percepts.

These three pairs of boundary and surface properties (inward vs. outward; oriented vs. unoriented; sensitive vs. insensitive to contrast polarity) are manifestly complementary.

**Figure 3:**
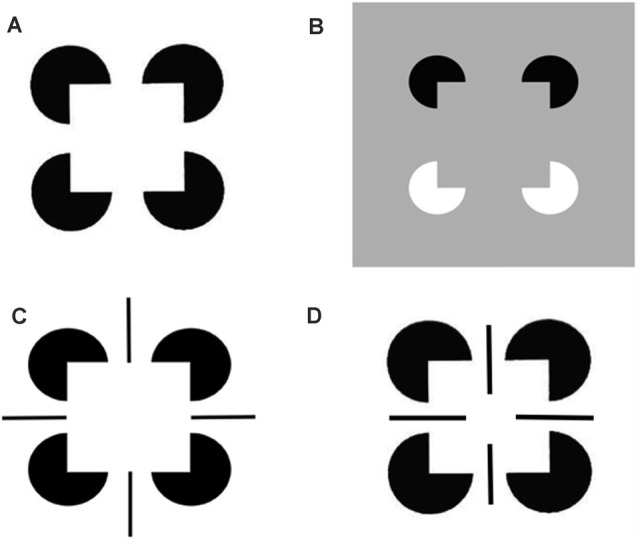
Kanizsa square and reverse-contrast Kanizsa square percepts: **(A)** The classical Kanizsa square is generated by four pac man figures with pairs of colinear edges. The Kanizsa square is perceived to be brighter than the white background because the black pac men induce brighter feature contours within the square due to the process whereby the illuminant is discounted. At a subsequent processing stage, the illusory contour boundaries that are induced by the square, and the feature contours that are adjacent to them, are both topographically projected. The feature contours can then fill-in the entire square with a higher level of perceived brightness. **(B)** A reverse-contrast Kanizsa square is induced by two black pac men and two white pac men on a gray background. The black pac men induce brighter feature contours, whereas the white pac men induce darker feature contours, than the gray background. When all the feature contours fill-in the emergent square, their contrasts cancel, leaving a percept of a gray square surface with the same contrast as that of the gray background. As a result, the Kanizsa square can be recognized but not seen. Panels **(C,D)** illustrate how edges and line ends can cooperate or compete to form illusory squares and disks in depth (Reprinted with permission from Grossberg, 2021).

The perceptual groupings that form due to boundary completion are sensitive to statistically significant spatial distributions of contour, texture, shading and depth cues in scenes and images (Julesz, 1971; Ramachandran and Nelson, 1976; Beck et al., 1983; Polat and Sagi, 1993). Illusory contours are a well-known type of perceptual grouping, and one which can be easily manipulated in the laboratory to reveal deep computational properties of the boundary completion process. Significantly, an illusory contour can form over image positions that do not receive contrastive bottom-up inputs from an image or scene. Figure 3 illustrates several examples of the illusory contours that induce percepts of Kanizsa squares (Kanizsa, 1955, 1974, 1976).

A surface representation is formed after the brain compensates for variable illumination conditions by discounting the illuminant, and then uses the surviving feature contours to fill-in surface qualities like brightness and color within completed boundary contours, or perceptual groupings. The percepts induced by the four images in Figure 3 illustrate how illusory surfaces with different perceived brightnesses and depths can form in response to different combinations of image inducers.

### 2.2. Spatial and Contrast Sensitivity of Kanizsa Square Percepts to Image Inducers

**Figure 4:**
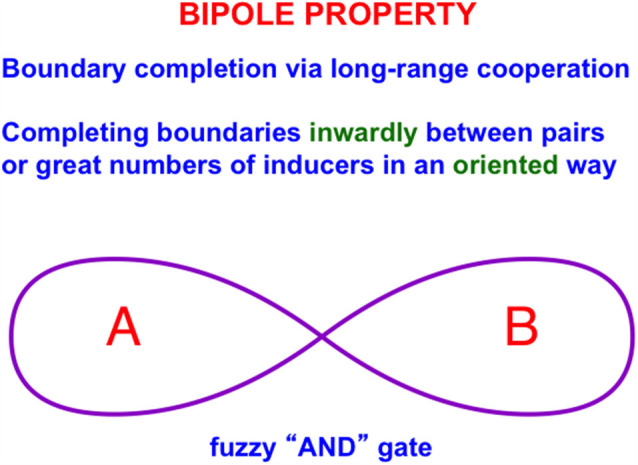
A bipole cell in layer 2/3 of cortical area V2 has two oriented branches, or poles, in its receptive field. The bipole cell can be activated by bottom-up inputs directly to its cell body. It can also be activated by sufficiently big inputs to both receptive field branches, hence the name “bipole” (Reprinted with permission from Grossberg, 2021).

Figure 3A illustrates the simplest kind of Kanizsa square, one that is induced by boundary completion between pairs of colinear edges of the four pac man figures. I have called this kind of inward boundary completion *bipole* boundary completion due to the fact that boundaries form inwardly between pairs of colinear inducers (Figure 4). Bipole boundary completion is realized in laminar neocortex using like-oriented and colinear horizontal axons in layer 2/3 of cortical area V2 (Grossberg, 1984a; Grossberg and Mingolla, 1985a, b). Similar, but shorter-range horizontal axons also exist in cortical area V1.

**Figure 5:**
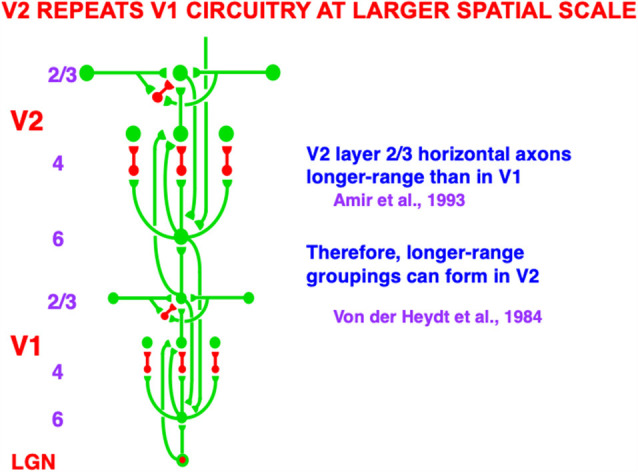
Macrocircuit of bottom-up, horizontal, and top-down pathways within and between the lateral geniculate nucleus (LGN) and cortical areas V1 and V2. The similarity of interactions in V1 and in V2 illustrates the canonical laminar neocortical circuit that occurs with one or another specialization in all perceptual and cognitive neocortical areas. The long-range horizontal connections in layer 2/3 of V1 and of V2 converge at bipole cells, with those in V2 of longer range than those in V1. Bipole cells in V2 are responsible for forming the boundary contours that generate illusory contours such as those displayed in Figure 3. Green connections are excitatory. Red connections are inhibitory (Reprinted with permission from Grossberg, 2021).

Said more concretely, a bipole cell in layer 2/3 of V2 in Figures 4 and 5 can fire under any of three conditions: (1) the bipole cell body—namely, the cell body that receives long-range horizontal connections from both sides—is directly activated by a bottom-up input from layer 4; (2) the cell body is not directly activated by a bottom-up input from layer 4, but it is simultaneously activated by signals from its long-range horizontal connections on both sides; or (3) the inputs in both (1) and (2) are simultaneously active.

In all the layers where oriented receptive fields develop within the V1 cortical map, horizontal interactions may also develop along with them, driven by a small number of developmental laws (Grossberg and Williamson, 2001). In addition to horizontal interactions among cells in layer 2/3, horizontal interactions may also occur between oriented simple cells in layer 4 (Sincich and Blasdel, 2001). The inhibitory horizontal interactions among simple cells whose development Grossberg and Williamson (2001) have modeled have been shown to play an important role in visual percepts of transparency (Grossberg and Yazdanbakhsh, 2005). Figure 3B shows an example of boundary completion in which the completed sides of the Kanizsa square can be consciously recognized but not seen. Such an invisible percept is sometimes said to be *amodal*. In both Figures 3A and 3B one can perceive that the pac man figures are amodally completed into circular disks “behind” the emergent Kanizsa square. Such depthful amodal percepts are even more vividly perceived in Figures 3C and 3D. Indeed, the ability to complete boundaries behind occluders in the natural world is crucially important to survival.

The percepts induced by Figure 3 illustrate how easily simple images can generate deep questions about how our brains consciously see. Since the 1980s, I and my colleagues have been incrementally developing a unified neural theory, called FACADE theory, to provide principled mechanistic explanations of how humans carry out 3D vision and figure-ground perception. The 3D LAMINART theory extends FACADE theory to explain how laminar cortical circuits naturally embody these perceptual processes, and to explain and predict much more psychological and neurobiological data as a result. My web page sites.bu.edu/steveg contains dozens of downloadable articles about the FACADE and 3D LAMINART theories that explain various aspects of how this happens. The following heuristic articles are a good place to start: Grossberg (1994, 1997).

## 3. Top-Down Attention Is Modulatory, Until Volition Activates Imagery, Thinking, Or Planning

### 3.1. Object Attention Is Focused by a Top-Down, Modulatory On-Center, Off-Surround Network

**Figure 6:**
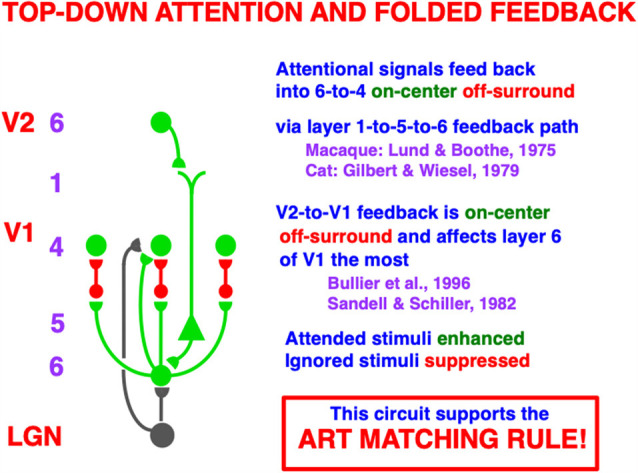
A top-down, modulatory on-center, off-surround network embodies how attention operates between cortical areas. Here a schematic of the circuit is shown from V2 to V1. A top-down signal from a cell in layer 6 of V2 activates a cell in layer 5 of V1 *via* its apical dendrites in layer 1. The layer 5 cell then activates a cell in layer 6 of V1 which, in turn, activates a modulatory on-center, off-surround network from layer 6-to-4 *via* “folded feedback”. This circuit embodies what is called the ART Matching Rule within Adaptive Resonance Theory. The ART Matching Rule dynamically stabilizes learned category memories within multiple brain systems. Green pathways are excitatory. Red pathways are inhibitory. The black pathways from LGN to layers 4 and 6 provide driving inputs to their target cells. When both direct inputs to layer 4 from LGN and indirect inputs from LGN to layer 4 *via* layer 6, converge upon a layer 4 cell, the resultant layer 4 activities are sensitive to the ratios of all the inputs received across the network. Contrast normalization is hereby achieved (Reprinted with permission from Grossberg, 2021).

Top-down attention enables humans to selectively process information that is of interest (Figure 6). In contrast to a perceptual grouping such as an illusory contour, top-down attention, acting by itself, does not form percepts over positions that receive no bottom-up inputs. Rather, attention can sensitize, modulate, or prime an observer’s mental state to expect an object to occur that has particular combinations of visual features (Duncan, 1984), or that is expected to occur at a given position (Posner, 1980). However, attention, acting by itself, cannot generate a consciously seen representation of an object. Figure 6 summarizes how object attention is embodied in laminar neocortex by a top-down, modulatory on-center, off-surround network that supports excitatory priming of features in the on-center, suppression of features in the off-surround, and gain amplification of matched data (e.g., Redies et al., 1986; Murphy and Sillito, 1987; Downing, 1988; Zeki and Shipp, 1988; Sillito et al., 1994; Steinman et al., 1995; Bullier et al., 1996; Hupé et al., 1997; Luck et al., 1997; Zhang et al., 1997; Caputo and Guerra, 1998; Roelfsema et al., 1998; Murphy et al., 1999; Reynolds et al., 1999; Somers et al., 1999; Mounts, 2000; Vanduffel et al., 2000; Temereanca and Simons, 2004; Reynolds and Heeger, 2009).

This kind of attentional circuit is said to obey the ART Matching Rule because of how it matches bottom-up inputs with top-down learned expectations within Adaptive Resonance Theory, or ART. It is easier to understand how this circuit works by considering it as part of ART, starting with a simplified version of it that is not encumbered by laminar cortical details. This is done in Section 3.2.

### 3.2. Three ART Matching Rule Functions: Learn Expectations, Pay Attention and Stabilize Memory

**Figure 7:**
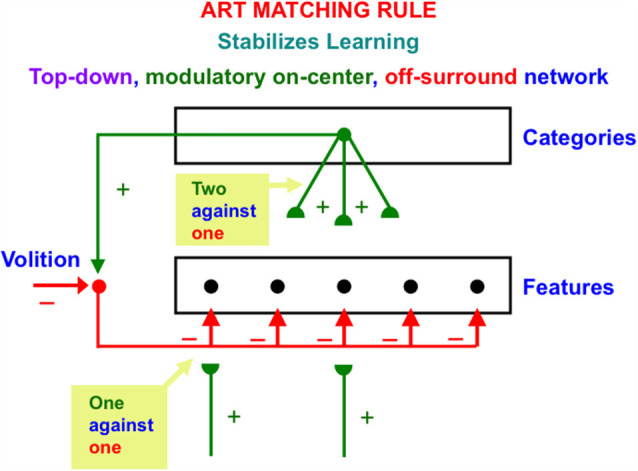
The ART Matching Rule stabilizes real time learning using a top-down, modulatory on-center, off-surround network. Object attention is realized by such a network. A volitional signal from the basal ganglia also enables humans to imagine, think, and plan. See the text for additional discussion (Reprinted with permission from Grossberg, 2021).

ART is currently the most advanced biological neural model of how our brains learn to attend, recognize, and predict objects and events in a changing world that is filled with unexpected events. This claim is based upon the fact that ART currently explains and predicts a much larger body of psychological and neurobiological data about these brain processes than alternative theories. See Grossberg (2013a, 2017, 2018, 2019, 2021) for reviews. Figure 7 depicts the ART Matching Rule circuit in a way that highlights its modulatory on-center and driving off-surround. In response to sufficiently large bottom-up excitatory inputs, the feature-selective cells at the lower processing level of Figure 7 can be driven to fire output signals, other things being equal. However, if only top-down signals from the category level are active, then feature-selective cells cannot fire. This is true because the top-down excitatory signals in the on-center (green pathways) are approximately balanced by top-down inhibitory signals in the off-surround (red pathways). This is a case of one excitatory signal balanced by one inhibitory signal. The net signal to feature-selective cells can sensitize, modulate, or prime them, but cannot fire them to suprathreshold levels.

Feature-selective cells can fire when they receive both bottom-up and top-down excitatory signals, because then two sources of excitatory signals converge in the on-center that can win over the inhibitory off-surround (two against one). Cells in the off-surround cannot fire, even if they receive a bottom-up excitatory input, because they are balanced by the top-down off-surround (one against one).

The adaptive weights, or long-term memory (LTM) traces in an ART Matching Rule top-down circuit can learn an expectation, or prototype, of *critical features* that are predictively important for classifying the object or event that the prototype encodes. These LTM traces occur at the ends, or synapses (denoted by green hemi-disks) of the top-down excitatory pathways in Figure 7. These expectations enable ART circuits to pay attention to their learned critical feature patterns. In addition to learning expectations and paying attention, the ART Matching Rule circuit is necessary to dynamically stabilize the recognition categories that ART circuits incrementally learn in response to the sequences of input patterns that they experience through time. In other words, the ART Matching Rule helps to solve the catastrophic forgetting problem. See Carpenter and Grossberg (1987a) for a mathematical proof, and the discussions of catastrophic forgetting throughout this article.

Figure 7 draws the off-surround overlapping on-center cells, rather than being restricted to cells outside the prototype. One reason for this is that it is not known before learning occurs what features will be part of the learned prototype. A no less important reason for this is that, in response to the first occurrence of a novel event, its bottom-up adaptive filter from the feature level to the category level must be able to learn a novel category, without the top-down signals that the category activates causing a mismatch at the feature level that could reset this category before it is learned. At the category level, however, it is not known what features are active at the feature level. The *initial* top-down expectation must be able to match *any* feature pattern. Thus, initial top-down expectation signals are uniformly large across all feature detectors. As category learning proceeds, the top-down expectation is pruned until it learns a prototype whose top-down signals are positive only at critical features.

Figure 7 also includes a volitional signal from the basal ganglia. Section 3.7 discusses how this volitional signal permits processes that are vital to the development of human societies, such as the ability to imagine, think, and plan.

### 3.3. High-Density Cortical Counterstream Architectures

Figure 5 summarizes key bottom-up, horizontal, and top-down cortical pathways within and between the LGN and cortical areas V1 and V2. These pathways are drawn from the perspective of a single LGN cell, or cell population, which sends outputs topographically to V1. The depicted circuit does not include the anatomically and functionally homologs top-down pathways from V1 to LGN, or the pathways between the LGN and the retina (Sillito et al., 1994; Gove et al., 1995; Grunewald and Grossberg, 1998; Murphy et al., 1999; Grossberg and Grunewald, 2002). However, the bottom image in Figure 12 does include feedforward and feedback pathways between LGN, V1, and V2.

**Figure 8:**
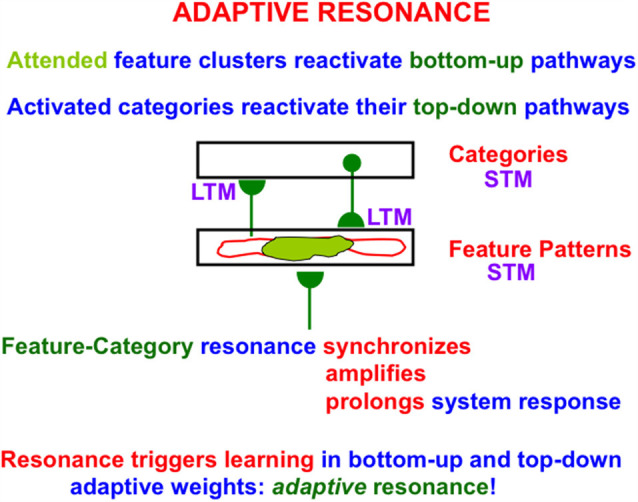
A feature-category resonance synchronizes, amplifies, and prolongs the activities of attended feature clusters (in light green), also called critical features, and the recognition category with which they are resonating via active bottom-up and top-down pathways. A feature-category resonance enables us to rapidly learn how to recognize objects without experiencing catastrophic forgetting. It hereby solves the stability-plasticity dilemma. Attentive matching between bottom-up feature pattern inputs and top-down expectations prevents catastrophic forgetting by focusing object attention upon expected critical features, while suppressing outlier features (outlined in red; Reprinted with permission from Grossberg, 2021).

**Figure 9:**
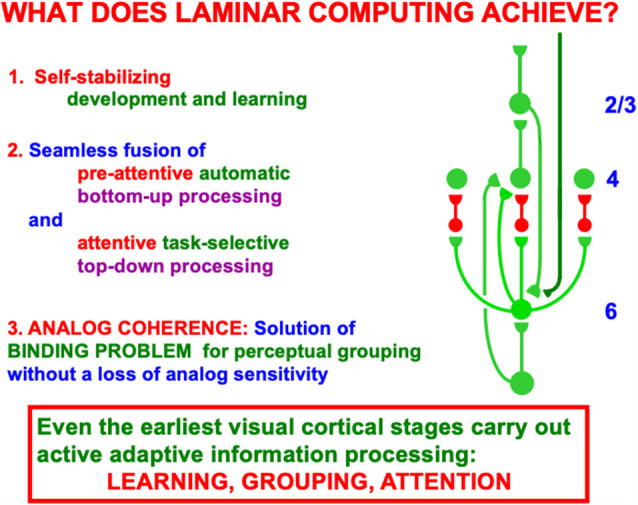
This figure summarizes some of the basic processes that are achieved when bottom-up and top-down inputs are processed within cortical layers 6 and 4. See the text for details (Reprinted with permission from Grossberg, 2021).

**Figure 10:**
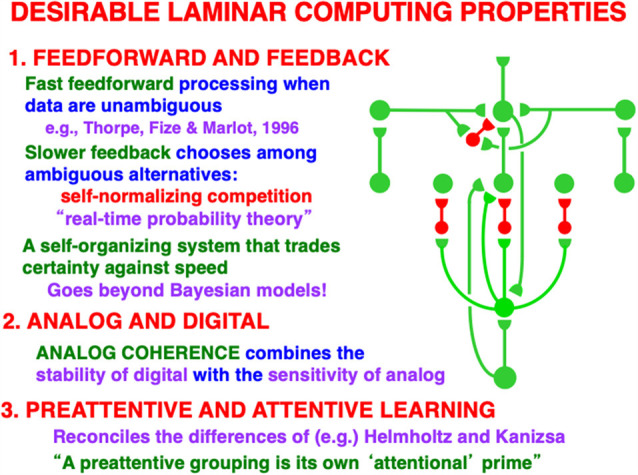
This figure summarizes some of the basic properties that are achieved when the bottom-up, horizontal, and top-down processes interact. The horizontal connections that support perceptual grouping, notably boundary completion, occur in layer 2/3. See the text for details (Reprinted with permission from Grossberg, 2021).

**Figure 11:**
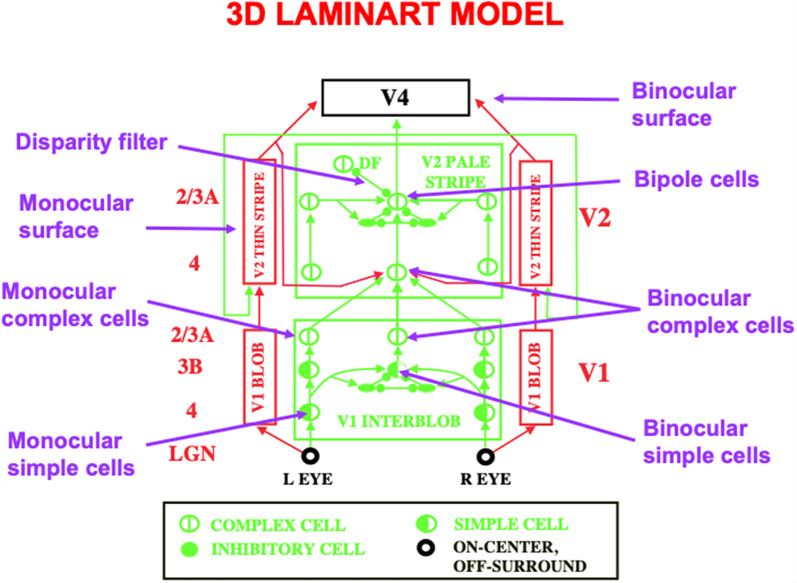
This schematic of the 3D LAMINART model labels some of the main cell types that are needed to achieve 3D vision and figure-ground perception. A 3D binocular surface percept in V4 is generated by a series of processing steps. Boundaries begin to be formed by monocular simple cells in layer 4 of V1. Monocular simple cells that respond to vertically oriented inputs with the same contrast polarity, but at horizontally displaced positions, add their outputs at binocular simple cells in layer 3B of V1 that respond selectively to different binocular disparities. Pairs of binocular simple cells that are sensitive to opposite contrast polarities at each position add their outputs at complex cells in layer 2/3A of V1. Complex cells input to bipole cells in layer 2/3 of V2. Interactions between boundaries and surfaces lead in successive stages to the binocular surface representation in V4, with monocular surfaces that fill-in brightnesses and colors from each eye separately in V2 before they are combined into the binocular surface representation in V4. Excitatory connections end in arrowheads. Inhibitory connections end in filled disks and ellipses (Reprinted with permission from Grossberg, 2021).

**Figure 12:**
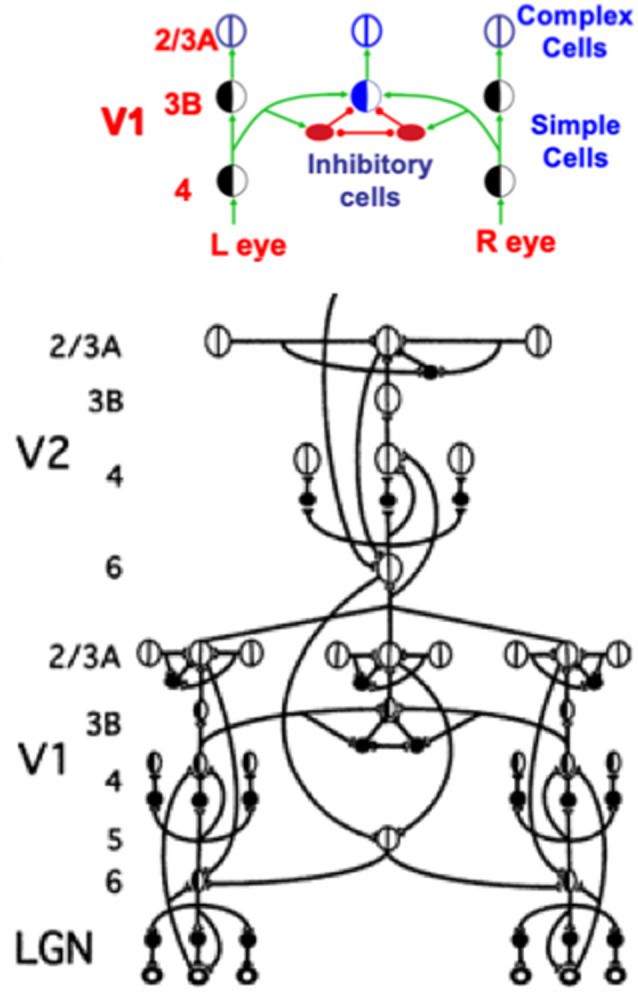
This figure shows how a balance between excitatory and inhibitory inputs from layer 4 to layer 3B creates disparity-selective binocular cells in layer 3B that obey an obligate property. See the text for details (Reprinted with permission from Grossberg, 2021).

A much greater omission occurs in Figure 5, one that is necessitated by the limitations of drawing topographically organized bottom-up, horizontal, and top-down pathways in a 2D picture. This omission is that the circuit in Figure 5 is repeated densely across LGN, V1, and V2. Indeed, the bottom-up pathways, taken together, and the top-down pathways, taken together, constitute “high-density counterstream architectures” (Markov et al., 2013; Vezoli et al., 2021).

As illustrated by the data of Barzegaran and Plomp (2021), one can tease apart “multiple concurrent feedforward and feedback streams in a cortical hierarchy” whose properties map upon the model circuits that are summarized herein. Taken together as a single slice through a dense cortical matrix of connections, Figures 5, 6 and 10 are consistent with their claim that there are “two feedforward and two feedback pathways” discernible in their data.

In fact, depending upon how one counts, which is limited by the resolution of particular experimental methods, there are more than two pairs of feedforward and feedback streams.

### 3.4. Form and Motion Stream Interactions Overcome Computationally Complementary Deficiencies

This fact becomes clearer when one supplements the primarily parvocellular cortical streams that subserve visual form perception through V1, V2, V4, and IT, among other cortical areas, with the primarily magnocellular parallel cortical streams that subserve visual motion perception through V1, MT, MST, and PPC, among other cortical areas. It has been shown that these parallel streams are needed because visual form and motion perception obey *computationally complementary laws* to accommodate the *orientationally-tuned* specializations needed for form perception and the *directionally-tuned* specializations needed for motion perception (Grossberg, 1991, 2014; Berzhanskaya et al., 2007).

Because visual form and motion are complementary, interactions across the form and motion streams are needed to overcome their complementary deficiencies, notably from V2 to MT, to compute moving-form-in-depth. The 3D FORMOTION model shows how form and motion information can be fused in MT by V2-to-MT interactions to enable efficient tracking of, say, an animal moving at variable speeds, even if its movement trajectory is intermittently occluded by trees, bushes, and other environmental obstructions (Francis and Grossberg, 1996; Baloch and Grossberg, 1997; Grossberg et al., 2001; Browning et al., 2009).

### 3.5. Complementary Computing: Are There Cortical Streams?

The V2-to-MT interaction links a ventral What cortical stream with a dorsal Where cortical stream. Figure 1 illustrates that form and motion are not the only computationally complementary pairs of cortical processing streams. The complementary properties of boundary completion and surface filling-in that are summarized in Figure 1 are overcome by interactions between the interblob and blob streams of the visual cortex, both of which are substreams of the What cortical stream.

Because complementary computing is basic design principle of our brains, there are many computationally complementary streams that need cross-stream interactions to overcome their complementary processing deficiencies (Grossberg, 2000b). Each cross-stream interaction mixes signals between the two streams, so that it is difficult for current neurobiological experimental methods to clearly measure their complementary properties. This general problem led Van Essen and Deyoe (1995, p. 383) in their review article in *The Cognitive Neurosciences* to question whether there are in fact cortical streams. They write: “Although there is widespread support for this hypothesis in general terms, it has proved difficult to decipher what exactly constitutes a visual processing stream and to ascertain the key functional differences between streams…”.

### 3.6. Feature-Category Resonance, Self-stabilized Memory, and Conscious Recognition

Figure 8 summarizes how the ART Matching Rule functions when the bottom-up adaptive filters and top-down learned expectations in ART architectures interact *via* their positive feedback loops (Figure 5). In Figure 8, a single pathway is drawn for simplicity to represent each array of bottom-up or top-down pathways. As noted in Section 3.2, the hemi-disk the hemi-disk synapses at the ends of these pathways are where learning can occur, or at the abutting postsynaptic membranes, or both. Learning in the bottom-up pathways tunes their adaptive weights, or long-term memory (LTM) traces, so that the cells which they activate respond selectively to particular combinations of environmental features. Such cells function as recognition *categories*. Activation of a category triggers read-out of top-down signals by pathways that obey the ART Matching Rule.

As in Figures 6 and 7 the ART Matching Rule on-center focuses attention upon critical features (the *attended feature clusters* colored light green in Figure 8) while cells in the off-surround are suppressed (the regions with a red outline).

When both the bottom-up and top-down pathways are activated by this positive feedback loop, their mutual activation synchronizes, amplifies, and prolongs the network response, leading to a *resonant* state. The particular type of resonance in Figure 8 is called a *feature-category resonance* because it binds together critical features and the categories that learn to code them. A feature-category resonance supports conscious recognition of the object or event that is coded by its critical feature pattern (Grossberg, 2017).

It is the feature-category resonance that drives an ART network to rapidly learn how to recognize objects, while also preserving learned memories indefinitely. Hence the name *Adaptive* Resonance Theory. In other words, ART networks do not experience *catastrophic forgetting*. Attentive matching between bottom-up feature pattern inputs and top-down expectations prevents catastrophic forgetting by focusing object attention upon expected patterns of features, while suppressing outlier features that might otherwise have caused catastrophic forgetting if they also were learned. In other words, the ART Matching Rule solves the catastrophic forgetting problem as part of resonant dynamics.

ART hereby solves the *stability-plasticity dilemma*; namely, how a system can learn quickly without also experiencing equally fast forgetting of an unpredictable part of its previously learned memories. See Grossberg (2020) for a discussion of why alternative neural models like back propagation and Deep Learning do experience catastrophic forgetting, and are thus untrustworthy models to use in applications.

ART dynamics have been used to explain how and where various brain oscillations occur—notably gamma, beta, and theta oscillations—and what their functional roles are in learning and behavior, including in the case of gamma and beta oscillations their laminar cortical distribution through time, and in the case of theta oscillations their cortical and subcortical generators. These explanations can be found in Grossberg and Versace (2008), Pilly and Grossberg (2013) and Grossberg (2017, 2021).

### 3.7. When ART Matching Is Not Modulatory: Volition, Imagery, Thinking, and Hallucinations

If attention is supplemented by volitional signals from the basal ganglia, it can be converted from a modulatory to a driving circuit that can activate target cells to suprathreshold values. A volitional signal from the basal ganglia can convert the modulatory on-center into a driving one by, for example, weakening the off-surround signal that inhibits excitatory on-center signals (Figure 7). How perceptual, cognitive, and motor processes are gated on and off by volitional signals from the substantia nigra pars reticulata (SNr) of the basal ganglia is reviewed in Grossberg (2016b).

Such a volitional signal enables us to internally perceive visual imagery (Kosslyn, 1996) if the top-down expectation that focuses attention has previously learned internal representations of those visual images. More generally, top-down expectations across a brain learn the featural information that is used to activate all of that brain’s recognition categories *via* feature-category resonances (Figure 8). The full diversity of top-down expectations can therefore be used to drive internal cognitive processes of thinking and planning that are essential to the success of human societies.

These benefits of volitional signals come with costs: if volitional signals become tonically hyperactive during a mental disorder, they can convert top-down attentional primes into hallucinations that represent vivid perceptual experiences that do not correspond to external stimuli. Grossberg (2000a) provides a unified explanation of various clinical data about hallucinations from this perspective, including why auditory hallucinations are typically more frequent than visual ones.

### 3.8. Contrasting ART With Bayesian Brain Models

The above-cited data properties are consistent with top-down ART circuits, but not with predictive coding models that embody Bayesian *explaining away*, in which matches with top-down feedback cause only suppression (e.g., Mumford, 1992; Rao and Ballard, 1999; Bastos et al., 2012). Bastos et al. (2012, p. 698) have, for example, proposed that “feedback connections convey predictions from higher cortical areas to suppress prediction errors in lower areas.” Their article cites several of the same articles that are cited herein. Indeed, all of these articles include evidence of top-down suppression or inhibition. Because the off-surround inputs are fully suppressive, whereas the on-center effects are merely modulatory, other things being equal, an impression of a fully suppressive top-down signaling may seem to exist. This impression can best be tested by doing experiments that additionally search for an on-center that can become driving in response to matched bottom-up inputs.

In addition to problems explaining the kinds of data described above, these models do not, at least currently, solve the stability-plasticity dilemma. Although ART is also a “predictive coding” model, because it instantiates its top-down predictive mechanisms using different principles and circuits than Bayesian models, it can solve the stability-plasticity dilemma, along the way explaining and simulating many experiments in which facilitatory on-center effects are reported. For a comparative discussion of biological vs. Bayesian approaches to understanding how our brains make perceptual decisions, see Grossberg and Pilly (2012) and Grossberg (2021).

## 4. A Solution of The Attention-Preattention Interface Problem

### 4.1. Attention and Grouping Both Occur Throughout the Visual Cortex

Perceptual groupings can form preattentively and automatically in cortical layer 2/3 using variants of bipole cells with long-range horizontal connections, even without conscious attention from the viewer, in both cortical area V1 (Redies et al., 1986; Grosof et al., 1993) and V2 (von der Heydt et al., 1984; Peterhans and von der Heydt, 1989), among other cortical areas, with the V2 groupings typically being longer-range (Figure 5). Top-down attentional circuits occur as early as from V1 to the lateral geniculate nucleus, or LGN (Sillito et al., 1994), as well as between pairs of all subsequent perceptual and cognitive neocortical areas (Figures 5 and 6). In particular, top-down attention operates between visual cortical areas V1, V2, and V4 that process visual form in the ventral, or What, cortical stream (Motter, 1994a, b; Reynolds et al., 1995; Beauchamp et al., 1997; Ito et al., 1997; Johnson and Burkhalter, 1997; Lamme et al., 1997; McAdams and Maunsell, 1997; Press and van Essen, 1997; Hupé et al., 1997), as well as between cortical areas MT, MST, and PPC that process visual motion in the dorsal, or Where, cortical stream (Treue and Maunsell, 1996; O’Craven et al., 1997).

### 4.2. Attention and Grouping Interact Throughout the Visual Cortex

I have called the interacting properties of attention and grouping the *attention-preattention interface problem*. Indeed, attention and grouping interact in all cortical areas, leading to my proposal that there are circuit *interfaces* where attentive and preattentive processes coexist. Figure 5 summarizes key anatomical interactions within and between layers 6 and 4 that define interfaces in both V1 and V2 where a top-down attentive process interacts with a horizontal preattentive grouping process.

### 4.3. Attention and Grouping Share a Modulatory On-Center, Off-Surround Circuit From Layer 6-to-4

As shown in Figure 5, V1 horizontal groupings form in layer 2/3 and send excitatory topographic signals to layer 6, where top-down attentional signals from layer 6 of V2 also project. This is where attentive and preattentive signals begin to comingle. Then layer 6 of V1 sends bottom-up signals to layer 4 using the kind of modulatory on-center, off-surround network that was discussed in Section 3 and Figure 6. Because top-down signals from V2 activate bottom-up signals from layer 6-to-4 in V1, this entire circuit is called a *folded feedback* circuit.

The on-center is created by excitatory topographic signals from layer 6-to-4. The on-center excitatory signal is reduced to being modulatory by inhibitory signals from layer 6-to-4 that are approximately the same size as the excitatory signals from layer 6-to-4, with the excitatory signals often slightly larger, as in Figures 6 and 7. The target layer 4 cells that receive these converging excitatory and inhibitory signals can thus be sensitized, modulated, or primed by them, but cannot fire to suprathreshold values in the absence of additional excitatory inputs. The off-surround inhibitory signals can strongly inhibit nearby layer 4 cells. This modulatory on-center, off-surround ART Matching Rule network is a design that is replicated in multiple cortical areas, as Figure 5 illustrates, in keeping with its proposed role in dynamically stabilizing learned receptive field properties in all cortical areas, as noted in Section 3.

At the time in 1998 that the model circuits in Figures 5 and 6 were submitted for publication, there were considerably fewer neurophysiological data to support model hypotheses than today. One significant exception were the data of Zeki and Shipp (1988) who wrote (p. 316) that “backward connections seem not to excite cells in lower areas, but instead influence the way they respond to stimuli,” in other words, they are modulatory.

In the late 1990s, more neurophysiological data were published to support this prediction. For example, Hupé et al. (1997, p. 1031) showed that “feedback connections from area V2 modulate but do not create center-surround interactions in V1 neurons,” and that top-down connections have an on-center off-surround organization (Bullier et al., 1996). An early hint that this on-center’s action is modulatory was reported by Sandell and Schiller (1982), who showed that, when feedback to V1 from V2 is eliminated by reversibly cooling V2, the V1 layer whose activation is most reduced is layer 6. Stratford et al. (1996) published compatible neurophysiological data showing that the layer 4 activation which is elicited by layer 6 stimulation is much weaker than that caused by stimulation of LGN axons or of neighboring layer 4 sites. These data also clarified how layer 4 cells could be supraliminally activated by direct LGN excitatory inputs, even when inputs from layer 6 were only modulatory, as I will explain below.

Taken together, these data show how attention from V2 to V1 may be realized by a top-down, modulatory on-center, off-surround network that projects from layer 6 in V2 to layer 4 in V1, often via apical dendrites of layer 5 cells in layer 1, which project to layer 6 cells in V1 (Lund and Boothe, 1975; Gilbert and Wiesel, 1979; Rockland and Virga, 1989) before undergoing folded feedback to end in a modulatory on-center, off-surround network from layer 6-to-4 (Figure 6). As part of Adaptive Resonance Theory, or ART, of which the 3D LAMINART theory is a laminar embodiment within the visual cortex, this kind of attentional circuit is said, as noted above, to obey the ART Matching Rule.

A more subtle kind of data from the 1990s showed that binocular layer 6 neurons input to monocular layer 4 cells of both eye types without reducing the monocularity of these layer 4 cells (Callaway, 1998, p. 56). These data can be explained by the facts that layer 6 neurons receive binocular inputs from layer 3B, at which monocular layer 4 inputs from both eyes converge on binocular cells, and that their top-down effect on layer 4 monocular cells is modulatory (Hubel and Wiesel, 1968; Poggio, 1972, 1991; Poggio and Fischer, 1977; Katz et al., 1989; Smith et al., 1997; Callaway, 1998; Grossberg and Howe, 2003; Cao and Grossberg, 2005, 2012, 2019). Section 5 will summarize how the canonical laminar circuit that is summarized in Figure 5 naturally accommodates these additional interactions to realize 3D vision and figure-ground perception.

The data of Sillito et al. (1994, pp. 479–482) on attentional feedback from V1 to LGN tell a similar story: “the cortico-thalamic input is only strong enough to exert an effect on those dLGN cells that are additionally polarized by their retinal input…the feedback circuit searches for correlations that support the “hypothesis” represented by a particular pattern of cortical activity.” Their experiments demonstrated all of the properties of a top-down, modulatory on-center, off-surround network from V1 to LGN, since they found in addition that “cortically induced correlation of relay cell activity produces coherent firing in those groups of relay cells with receptive-field alignments appropriate to signal the particular orientation of the moving contour to the cortex…this increases the gain of the input for feature linked events detected by the cortex.” In other words, top-down priming, by itself, cannot fully activate LGN cells. Matched bottom-up retinal inputs are needed to do so. Those LGN cells whose bottom-up signals support cortical activity get synchronized and amplified by this feedback.

Anatomical studies have also supported these neurophysiological conclusions by showing that the top-down V1-to-LGN pathway realizes a top-down, on-center, off-surround network (Dubin and Cleland, 1977; Weber et al., 1989).

### 4.4. LGN Inputs to Layers 4 and 6 Drive Normalized Cortical Responses at Layer 4

The previous discussion describes various overlapping pathways between attention and grouping within the laminar circuits of V1 and V2. However, they do not explain how these circuits get activated to suprathreshold values by external inputs. Figure 6 provides an answer to this question. The bottom-up pathways (in black) from the LGN to layers 4 and 6 are both excitatory. Although the layer 6-to-4 on-center off-surround circuit can only modulate the activities of cells in layer 4, the direct LGN input to layer 4 can, by itself, drive layer 4 cells to fire at suprathreshold values.

Given that the LGN-to-4 pathway can fire layer 4, why is there also an LGN-to-6 pathway that can modulate layer 4 activity via the layer 6-to-4 on-center off-surround network? Were it to act alone, the LGN-to-4 pathway could *saturate* the responses of layer 4 cells at their maximum values. Even small increases in the LGN input could, in principle, do this. The dynamic range of analog sensitivity to changing input amplitudes would then be quite small.

If, however, LGN inputs to both layer 4 and layer 6, then the total input to layer 4—when one adds the direct input from LGN and the indirect LGN input *via* layer 6—defines a *driving* on-center, off-surround network. All the cells in this laminar cortical architecture obey the membrane equations of neurophysiology, also called shunting laws (Hodgkin and Huxley, 1952). In a network whose cells obey shunting laws, it has been mathematically proved that responses of layer 4 cells to LGN inputs via an on-center off-surround network remain sensitive to the *ratios* of their inputs—that is, to their *spatial pattern*—even if the total input amplitude changes greatly through time (Grossberg, 1973, 2013b).

By causing cell responses to track the ratios of their inputs, it follows that the *total* network activity tends to be *normalized*; that is, its upper bound is independent of the number of activated cells. The responses of layer 4 cells thus exhibit *contrast normalization*. Such a shunting on-center off-surround network is said to solve the *noise-saturation dilemma* because its inputs can be chosen large enough to be registered accurately despite internal cellular noise, without risking that they will be chosen so large to cause cell activities to saturate (Grossberg, 2013b).

### 4.5. What Does Laminar Computing Achieve? Consensus Between Bottom-Up, Horizontal, and Top-Down

When both bottom-up driving inputs and top-down, modulatory on-center, off-surround attentional signals come together, the ensuing network realizes a combination of useful properties. As summarized in Figure 9, this network is:

(1) Self-stabilizing in the sense that it can dynamically stabilize memories formed by development and learning processes. Said in another way, the network does not experience catastrophic forgetting (Grossberg, 2020, 2021). These ART learning properties that rely on the ART Matching Rule are mathematically proved in articles such as Carpenter and Grossberg (1987a). The network also realizes:
(2) A seamless fusion of automatic, preattentive, data-driven, bottom-up processing and attentive, task-selective, top-down processing within the on-center off-surround layer 6-to-4 circuit that combines these constraints and chooses the best possible consensus of them to control network decision-making. Finally the network can do this while maintaining:
(3) *Analog coherence*, or a solution of the binding problem for perceptual grouping without a loss of analog sensitivity. Said in another way, the network solves the noise-saturation dilemma.

### 4.6. Unifying Feedforward-Feedback, Analog-Digital, and Preattentive-Attentive Learning Constraints

Figure 10 restores the horizontal grouping network to the bottom-up and top-down processing circuits in Figure 9. Figures 5, 9 and 10 each show how bottom-up, horizontal, and top-down circuits can all influence the competitive decision making process in layers 6-to-4, leading to the best consensus at any time of these distinct processing constraints.

Another way to say how bottom-up, horizontal, and top-down pathways cooperate during decision making is as follows: the bottom-up inputs instate distributed spatial patterns of feature categories in a scene. The horizontal interactions preattentively bind these feature categories into emergent object boundary representations. The top-down interactions read-out familiar object boundary representations and use the ART Matching Rule to select, gain amplify, and synchronize object boundaries that fall within their modulatory on-center, while suppressing object boundaries that fall within their off-surround. These interactions can hereby create novel preattentive object boundaries across a scene while they attentively select the familiar ones that are of current interest upon which to base decisions and predictions.

This kind of canonical circuit, suitably specialized, occurs in essentially all laminar perceptual and cognitive neocortical areas, not only the visual ones. Raizada and Grossberg (2003) summarize data from a large number of experiments that support every cell and pathway in this circuit. As summarized in Figure 10, this canonical neocortical circuit parsimoniously unifies several combinations of computational properties that are not easy to reconcile in a single system:

### 4.7. Feedforward and Feedback

In response to unambiguous inputs, a fast feedforward sweep of activation can progress throughout multiple cortical areas, as Thorpe et al. (1996) previously demonstrated. If, however, there are multiple ambiguous alternative interpretations of the data, then the kind of self-normalizing competitive feedback processing that Figure 10 illustrates can more slowly choose the interpretation with the currently greatest support from these combined constraints.

Self-normalizing ratio processing can be interpreted as a kind of “real-time probability theory” (Figure 10). When ART hypothesis testing and memory search for the best alternative is added to the mix, then this “probability theory” is embedded within a self-organizing production system that incrementally learns, using arbitrary combinations of unsupervised and supervised learning, to rapidly classify large non-stationary databases without experiencing catastrophic forgetting (Carpenter and Grossberg, 1987a; Grossberg, 2020, 2021). ART hypothesis testing trades certainty against speed, with the most unambiguous data leading to the fastest categorizations, decisions, and predictions.

### 4.8. Analog and Digital

Again using vision as an example: recurrent shunting on-center off-surround networks are capable of binding distributed features within coherent boundary representations without a loss of analog sensitivity. This property of *analog coherence* combines the *stability* of digital processing—because the feedback signals store the chosen representation and buffer it against noisy perturbations—and the *sensitivity* of analog processing—because the shunting on-center off-surround interactions solve the noise-saturation dilemma.

### 4.9. Preattentive and Attentive Learning

The circuit depicted in Figures 9 and 10 shows how both preattentive grouping and attentive matching *via* the ART Matching Rule mutually influence one another. The current discussion focuses primarily on the circuit *per se*. A more sustained analysis is needed to explain how ART uses the ART Matching Rule to dynamically stabilize category learning in all perceptual and cognitive neocortical areas (Carpenter and Grossberg, 1987a, b; Grossberg, 1987, 1988, 1995, 2003, 2013a, 2021). ART hereby overcomes the catastrophic forgetting problem that essentially all other popular neural models experience, notably the currently popular Deep Learning model (Grossberg, 2020), which uses the back propagation learning law and thus suffers from 17 problems that are not problems for ART (Grossberg, 1988).

### 4.10. A Preattentive Grouping Is Its Own Attentional Prime

ART Matching Rule because it is typically realized in larger ART neural architectures that learn how to attend, recognize, and predict objects and events in a changing world. A basic result about this learning process was explained in Carpenter and Grossberg (1987a) who also proved mathematically that the ART Matching Rule prevents learned recognition categories from experiencing catastrophic forgetting. In other words, attentional matching dynamically stabilizes learned memories.

However, when the ART Matching Rule is removed, then it is easy to construct environments in which catastrophic forgetting does occur. These environments, moreover, can be quite simple. For example, learning categories of just four input patterns (A, B, C, D) can cause catastrophic forgetting when the inputs are presented over and over in the order ABCAD ABCAD etc., *if* the input patterns obey some simple constraints. When these constraints hold, catastrophic forgetting occurs because input pattern A is periodically classified by two different categories, so its learned category never stabilizes. Grossberg (2020) summarizes this result from Carpenter and Grossberg (1987a) as well as the efforts of recent models to overcome their catastrophic forgetting problems.

In order for the ART Matching Rule to dynamically stabilize learned memories, its top-down on-center must be *modulatory*. In other words, bottom-up inputs must occur in order to fire cells that are modulated by attention. Illusory contours seem to violate this constraint because, by their very nature, they complete suprathreshold boundaries over positions that receive no bottom-up inputs (Figure 3). It is also known that the cells and connections whereby perceptual boundaries form develop through experience-dependent learning (Hubel and Wiesel, 1977; Stryker and Harris, 1986; Calloway and Katz, 1990; Antonini and Stryker, 1993; DeAngelis et al., 1993; Ghose et al., 1994; Durack and Katz, 1996; Galuske and Singer, 1996; Ruthazer and Stryker, 1996; Sur and Learney, 2001). Since these learned connections support the formation of illusory contours, they seem to violate the ART Matching Rule. Why, then, are they not degraded regularly by catastrophic forgetting?

My proposed solution to this problem is that both preattentive grouping and attention use the same competitive decision circuit within layers 6 and 4 as part of the attention-preattention interface (Figures 9 and 10). This modulatory on-center, off-surround network in both networks dynamically stabilizes their learning, whether in response to inputs from layer 6 of a higher cortical area in the case of attention, or from layer 2/3 in the same cortical area in the case of boundary grouping.

Due to the latter property, I like to say that “a preattentive grouping is its own attentional prime.” From a broader perspective, this result highlights the functional wisdom that is embodied within the feedforward and feedback interactions that have been selected by evolution to carry out dynamically stabilized learning during both attentive and preattentive processing.

## 5. 3D Laminart: Canonical Circuit Generalizes to 3D Vision and Figure-Ground Perception

### 5.1. Extra Neocortical Layers Enable 3D Vision to Occur

Multiple refinements of the canonical laminar cortical circuit are needed to enable 3D vision and figure-ground perception to occur. The labels in the 3D LAMINART circuit of Figure 11 highlight some of the processes that are needed. These refinements are introduced and explained in detail in FACADE theory and its 3D LAMINART generalization (e.g., Grossberg, 1994, 1997, 2016a; Grossberg and McLoughlin, 1997; Kelly and Grossberg, 2000; Grossberg and Howe, 2003; Grossberg and Swaminathan, 2004; Cao and Grossberg, 2005, 2012, 2019; Grossberg and Yazdanbakhsh, 2005; Grossberg et al., 2008; Fang and Grossberg, 2009). Remarkably, they can all build upon the canonical neocortical circuit that has already been summarized.

For present purposes, one main point will be made as an answer to the following basic question: in what V1 cortical layer do cells first become binocular, and how do the layers of the canonical circuit permit this refinement?

### 5.2. Binocular Disparity-Selective Cells Occur First in Layer 3B

The answer is that binocular matching begins within a layer 3B that is interpolated between layers 4 and 2/3 in V1 (Figure 12). Layer 4 contains monocular simple cells that respond at a particular position and orientation to a visual stimulus to either the left or right eye, but not both. Layer 2/3 in V2 is where the kind of boundary grouping occurs that can generate illusory contours. Because groupings can form between an object’s image features with opposite contrast polarities (e.g., Figure 3B), the cells in layer 2/3 are downstream from complex cells that can pool inputs from opposite contrast polarity features (Figures 2 and 12). Groupings can also occur between object features at different depths (e.g., Figure 3D). Binocular fusion thus needs to occur between layers 4 and 2/3 in V1 in order for perceptual groupings to represent objects in depth.

In layer 3B, pairs of like-oriented inputs from positionally-displaced left and right eye monocular simple cells of similar orientational preference in layer 4 are binocularly matched (Figure 12). If only excitatory inputs were matched, then these binocular cells would not fire *only* if their left and right eye inputs were *both* activated, and activated approximately equally due to the fact that the same feature in the world has activated them.

This *obligate property* (Poggio, 1991) is realized by letting the excitatory inputs to layer 3B also activate inhibitory interneurons which inhibit both their layer 3B target cells and each other (Figure 12, upper panel). The mutual inhibition normalizes the total activity of the inhibitory interneuron population. As a result, if only (say) the left eye is activated, then its excitatory input to layer 3B will be inhibited by an approximately equal inhibitory input from its recurrent inhibitory interneuron. If, in contrast, *both* the left and right eyes are activated, then their total excitatory inputs to their layer 3B target cell add, whereas the total inhibitory input to the target layer 3B cells from all the active inhibitory interneurons is normalized. This is a case of two excitatory inputs balanced against one inhibitory input (2 against 1), so that the target layer 3B cell can then fire. It is thus a truly binocular cell which fires selectively only when the depth of the object in the world creates a binocular disparity that can activate both the left and right eye inputs to the corresponding disparity-selective layer 3B cells.

### 5.3. Binocular Boundary Formation Circuitry From LGN to V1 and V2

As noted in Section 4.3, a binocular cell in layer 3B is part of a feedback loop that sends top-down signals to monocular simple cells in layer 4, and whose effect on layer 4 monocular cells is modulatory. This modulatory effect can now be understood as a special case of the ART Matching Rule for dynamically stabilizing the learned binocular fusion at layer 3B cells of V1 that ensures their disparity selectivity.

The cells in layer 3B where binocular fusion occurs are binocular simple cells. These binocular simple cells combine inputs from both the left eye and the right eye. When both eyes fixate on a particular part of an object, light signals from every other part of the object hit the two eyes at different positions relative to their foveas. These positionally-shifted signals activate monocular simple cells at different, but nearby, positions in the visual cortex. The outputs from these monocular simple cells are pooled at binocular simple cells in layer 3B that lie at an intermediate position between these monocular simple cells in the cortical map. This first stage of binocular processing enables the brain to begin estimating the depth of an object relative to the observer using the size of this positional difference, which is called *binocular disparity.* Different binocular simple cells, and the binocular complex cells in layer 2/3A to which they project (Figures 11 and 12), hereby become sensitive to different ranges of binocular disparity, and thus to different depths of objects from the observer.

Thus, because the cells in layer 3B binocularly fuse signals from monocular simple cells that respond best at disparate positions, the binocular boundaries that start to be formed in layer 3B, and their projections to layers 2/3A in V1 and V2 (Figures 11 and 12), may be positionally displaced, or shifted, relative to their monocular input signals from layers 6 and 4. This raises the question of how the positionally displaced binocular boundaries in layer 2/3A of V2 manage to contact the correct monocularly activated cells in layers 6 and 4, so that they can complete the ART feedback loop between layers 2/3A-to-6-to-4-to-3B-to-2/3A that can select winning 3D groupings.

Figure 12 (upper panel) shows how horizontal signals from the monocular layer 4 cells in V1 activate binocular obligate cells in layer 3B, which in turn activate layer 2/3A complex cells. This raises the question: How can such a layer 2/3A cell also use horizontal signals to activate its correct layer 6 monocular sources via ART Matching Rule circuits? The 3D LAMINART model (Grossberg and Howe, 2003; Grossberg, 2003) proposes that horizontal connections which are known to occur in layers 5 and 6 (Callaway and Wiser, 1996) accomplish this. As shown in Figure 12 (lower panel), feedback signals from layer 2/3A propagate vertically to layer 5, whose cells activate horizontal axons in this layer that contact the appropriate layer 6 cells. These layer 5-to-6 horizontal contacts are assumed to be selectively formed during development. Grossberg and Williamson (2001) and Grossberg (2003) have simulated how positionally-aligned layer 2/3 connections and layer 6-to-4 connections may be formed during development. The selective layer 5-to-6 contacts are proposed to form according to similar developmental laws.

In summary, inward horizontal layer 4-to-3B and 2/3A-to-2/3A connections are proposed to form binocular cells and their boundary groupings, respectively, while outward layer 5-to-6 connections are proposed to close the feedback loops that help to select and sustain the correct 3D groupings via ART learning dynamics. This role in 3D grouping of layer 6 horizontal connections forces a proposal for how attention fits into the 3D circuit: namely, top-down topographic signals from layer 6 of a higher cortical level like V2 activate the same layer 5 cells that contact monocular input sources in layer 6 via horizontal connections. Then the layer 6-to-4 modulatory on-center, off-surround network controls attentional priming and matching, as in Figure 6.

This hypothesis raises the additional question of how the top-down pathways from layer 6 of a higher cortical level know how to converge on the same layer 5 and 6 cells to which the layer 2/3 cells project at the lower cortical level so that preattentive boundary grouping and attentive priming are positionally aligned (Figure 9). The same basic laws of associative learning are proposed to work here as well.

### 5.4. Homologous Circuits in V1 for Binocular Fusion and in V2 for Perceptual Grouping

It is worth noting that the bipole cells which control boundary completion also use a “2 against 1” network of excitatory and inhibitory interactions to form boundaries inwardly between pairs of colinear inducers, but not outwardly from a single inducer. Figure 11 illustrates these homologous circuits in layer 3B of V1 and layer 2/3A of V2.

Just as in the case of binocular fusion, if a single cell in layer 2/3 of V2 sends an excitatory signal to a nearby cell, then its excitation is balanced by inhibition from a recurrent inhibitory interneuron. If, however, a pair of colinear cells activate a similarly oriented cell between them, their excitatory inputs add, but their total inhibitory input is normalized by recurrent inhibitory interactions between their inhibitory interneurons. In summary, homologous combinations of excitatory and recurrent inhibitory interactions occur in layer 3B of V1 to support binocular fusion, and in layer 2/3A of V2 to support bipole boundary grouping, respectively (Grossberg and Williamson, 2001).

A third homologous circuit exists in layer 2/3A of V1 to carry out the shorter-range version of the bipole grouping that is shown in layer 2/3A of V2. This third circuit is omitted from Figure 11 to avoid crowding. It is included in Figure 5.

## 6. List Parse: Canonical Circuit Generalizes to Working Memory and List Chunks

### 6.1. A Universal Design for Linguistic, Spatial, and Motor Working Memories

Specializations of the canonical laminar cortical design are found throughout the neocortex in modalities other than vision. The LIST PARSE laminar cortical model of working memory in prefrontal cortex is one example. LIST PARSE is able to store sequences of variable length and to perform them at variable speeds under volitional control. LIST PARSE is said to be an Item-Order-Rank working memory because it can temporarily store sequences of *item* chunks in a particular *order*, even for lists such as ABACBD that contain repeated items, such as A and B, at more than one list position, or *rank*. It is a *content-addressable* working memory because inputs directly activate item chunks.

The items that are stored with the largest activities are performed soonest. This happens because, in response to a rehearsal wave that is nonspecifically, and thus equally, delivered to all the item chunks, the cell whose activity is largest exceeds its output threshold first. As each cell fires, it self-inhibits its stored activity so that the next most active cell can be performed. This cycle continues until all the items that are stored in working memory have been rehearsed (Grossberg, 1978a, b; Grossberg and Pearson, 2008).

LIST PARSE working memories are used in storing all kinds of linguistic, spatial, and motor sequences. It is a ubiquitous design in multiple brain regions because it enables learning and stable memory of these sequences for future skilled performance and prediction of subsequent events. For example, LIST PARSE models a prefrontal *linguistic* working memory to quantitatively simulate psychophysical data about speech perception, immediate serial recall, and immediate, delayed, and continuous distractor free recall (Grossberg and Pearson, 2008; Grossberg and Kazerounian, 2011). LIST PARSE models a prefrontal *motor* working memory to quantitatively simulate neurophysiological data about sequential recall of stored motor sequences, as when performing a dance or other skilled motor act (Grossberg and Pearson, 2008). Finally, LIST PARSE models a prefrontal *spatial* working memory to quantitatively simulate neurophysiological data about the learning and planned performance of saccadic eye movement sequences or other spatially represented activities (Silver et al., 2011).

**Figure 13:**
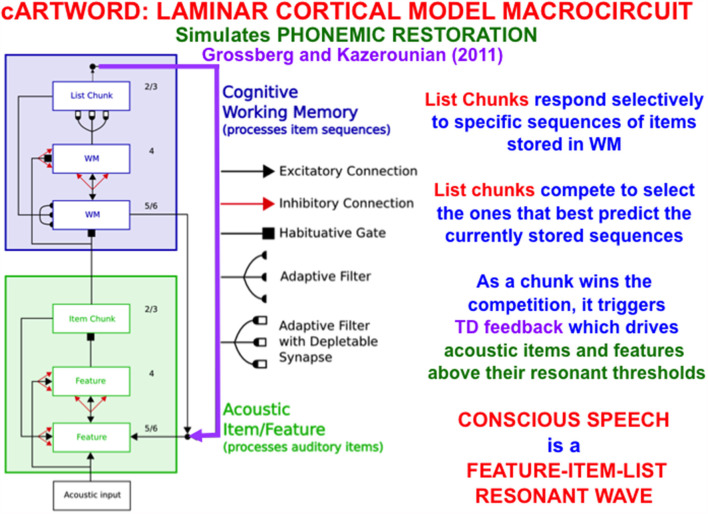
The cARTWORD model of conscious speech perception ends in an Item-Order-Rank laminar cortical cognitive working memory (WM) whose list chunks can store sequences of linguistic items with repeats. As a list chunk in layer 2/3 of the cognitive working memory wins the competition among other chunks, it triggers top-down feedback (vertical downward purple arrow) that drives acoustic items and features above their resonant thresholds. cARTWORD hereby simulates percepts of *phonemic restoration*; namely, how future context can disambiguate noisy past speech sounds in such a way that the completed percept is consciously heard to proceed from past to future as a feature-item-list resonant wave propagates through time (Reprinted with permission from Grossberg, 2021).

In order to carry out such linguistic, motor, or spatial functions, a LIST PARSE working memory is just part of a larger neural architecture. Figure 13 summarizes the macrocircuit of the *conscious ARTWORD*, or cARTWORD, architecture for conscious speech perception that uses LIST PARSE to temporarily store sequences of linguistic items, supplemented by auditory preprocessing stages that transform acoustic inputs into the item chunks that input to working memory (Grossberg and Kazerounian, 2011, 2016; Kazerounian and Grossberg, 2014).

### 6.2. Masking Fields Learn List Chunks of Stored Item Chunk Sequences

**Figure 14:**
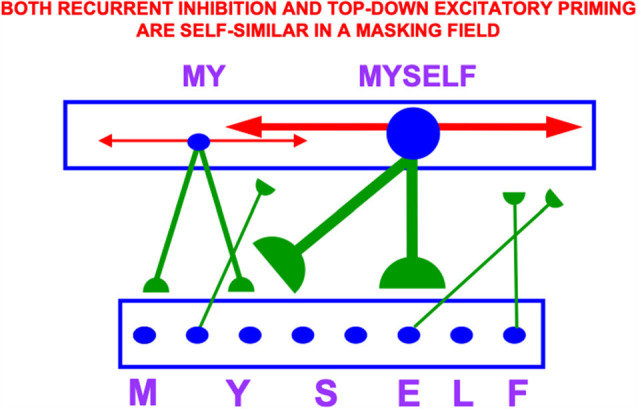
A Masking Field is a recurrent shunting on-center off-surround network whose cells can learn categories that selectively fire to sequences, or lists, of items that are stored in an Item-Order-Rank working memory. In order to selectively fire in response to stored lists of multiple lengths, Masking Field cells and their connections develop with multiple sizes according to simple activity-dependent growth rule (Cohen and Grossberg, 1986; Grossberg, 1987), with the largest cells responding to the longest lists. These multiple scales are related to each other through a property of self-similarity, which enables a Masking Field to choose the category that best represents the sequence that is currently stored in working memory. Thus a cell in the Masking Field that selectively responds to the word MYSELF is larger than a cell that responds to MY. Both its top-down adaptive connections (in green with hemi-disks at their ends) and its recurrent inhibitory connections (in red with non-adaptive arrows at their ends) have a strength and size that covaries with that of the cell from which they grow by self-similar growth laws. Self-similar growth is familiar throughout biological development, much as a small leaf can grow into a larger leaf without dramatically changing its shape (Reprinted with permission from Grossberg, 2021).

Item-Order-Rank working memories have many desirable properties, including their ability to support learning and stable memory of categories that respond selectively to particular sequences of stored items, such as familiar syllables, words, and sentences. These sequence categories are also called *list chunks*. List chunks of variable length can be categorized by a multiple-scale, self-similar, recurrent, shunting, on-center off-surround network that is called a Masking Field (Figure 14).

The LTM Invariance Principle is the main postulate from which Item-Order-Rank working memories are derived. The LTM Invariance Principle insists that working memories be *designed* to enable stable learning of list chunks by a network like a Masking Field.

The LTM Invariance Principle guarantees, for example, that the first time a novel word, such as MYSELF, is stored in working memory, it does not force the forgetting of previously learned list chunks that code for its familiar subwords MY, ELF, and SELF. Without such a property, longer chunks (e.g., for MYSELF) could not be stored in working memory without risking the catastrophic forgetting of previously learned memories of shorter chunks (e.g., for MY, SELF, and ELF). Language, motor, and spatial sequential skills would then be impossible. As a consequence of LTM Invariance, as new items are stored through time in working memory, smaller stored sequences of items can continue to activate their familiar list chunks until they are inhibited by longer, and more predictive, list chunks; e.g., until MY is supplanted by competition from MYSELF through time (Figure 14).

The LTM Invariance Principle is achieved by preserving the *relative activities*, or ratios, between previously stored working memory activities as new list items are stored in the working memory through time. Newly arriving inputs may, however, alter the *total activity* of each active cell across the working memory. In this way, the previous *learning* of chunk MY is not undermined, but the *current activity* of the chunk MY can be inhibited by MYSELF.

Masking Fields have been used to explain and simulate psychophysical data from many experiments about how speech sounds are temporarily stored in an Item-Order-Rank working memory before they are categorized through learning, and recognized during an *item-list resonance* with a Masking Field (Grossberg, 1984b, 2003, 2017; Cohen and Grossberg, 1986, 1987; Grossberg and Stone, 1986; Cohen et al., 1988; Grossberg et al., 1997; Grossberg and Myers, 2000; Grossberg and Kazerounian, 2011; Silver et al., 2011; Kazerounian and Grossberg, 2014). The Masking Field can then, in turn, predict appropriate outcomes in the temporal context of the event sequence that its categories encode, whether they be the next words, visual scenes, eye movements, arm movements, or navigational movements.

### 6.3. pART: A Neural Architecture to Clarify How Autonomous Adaptive Biological Intelligence Works

The ability of an Item-Order-Rank working memory to store the same item at multiple positions derives from a topographic projection from the parietal cortex to the ventrolateral prefrontal cortex. This projection converts numerical representations in the parietal cortex (Dehaene, 1992, 1997; Grossberg and Repin, 2003) into the ranks of items stored in prefrontal working memory (Barone and Joseph, 1989; Averbeck et al., 2003a, b; Mushiake et al., 2006; Grossberg and Pearson, 2008; Silver et al., 2011), so that items in stored sequences with repeats each have their own positional representations in prefrontal hypercolumns. Remarkably, just two successive levels of Item-Order-Rank working memories can store sequences of repeated words, as in the sentence “DOG EATS DOG” (Grossberg, 2021).

**Figure 15:**
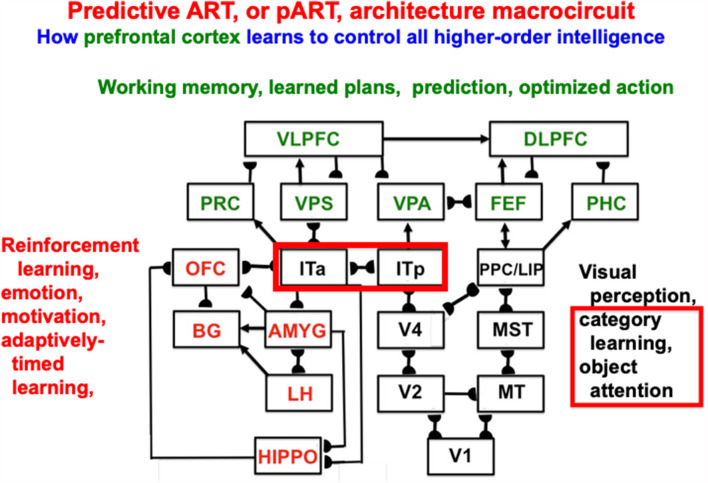
Macrocircuit of the predictive ART, or pART, architecture which integrates key processes that are needed to achieve biological intelligence. The various brain regions are color-coded along with their functional roles in brain dynamics. The architecture illustrates that many higher-order functions can be carried out with a relatively small number of brain regions, if they are properly designed. Abbreviations: V1, striate, or primary, visual cortex; V2 and V4, areas of prestriate visual cortex; MT, middle temporal cortex; MST, medial superior temporal area; ITp, posterior inferotemporal cortex; ITa, anterior inferotemporal cortex; PPC, posterior parietal cortex; LIP, lateral intraparietal area; VPA, ventral prearcuate gyrus; FEF, frontal eye fields; PHC, parahippocampal cortex; DLPFC, dorsolateral prefrontal cortex; HIPPO, hippocampus; LH, lateral hypothalamus; BG, basal ganglia; AMGY, amygdala; OFC, orbitofrontal cortex; PRC, perirhinal cortex; VPS, ventral bank of the principal sulcus; VLPFC, ventrolateral prefrontal cortex (Reprinted with permission from Grossberg, 2021).

Item-Order-Rank working memories illustrate the general hypothesis that a relatively small number of processing stages are needed to realize many properties of biological intelligence. In particular, these working memories form part of the predictive Adaptive Resonance Theory, or pART, neural architecture which also includes other processes that are needed to explain how our brains make our minds (Grossberg, 2018, 2021). Figure 15 illustrates, for the case of visual intelligence, how four collections of brain regions interact to realize this goal within pART. The working memories combine with learned plans, predictions, and optimized actions as part of a system of seven prefrontal cortical areas that are marked in green. Processes that are marked in red carry out evaluative processes that modulate and regulate the prefrontal processes using reinforcement learning, emotion, motivation, and adaptively-timed learning. The prefrontal processes are fed by category learning and recognition circuits in the posterior inferotemporal cortex (Itp) and the anterior inferotemporal cortex (Ita; outlined with a red border), as well as by spatial representations from the posterior parietal cortex (PPC/LIP), so that predictions may be based both on objects and their locations. The categories receive their inputs from multiple visual cortical areas, marked in black, that interact to enable our brains to see.

## 7. Concluding Remarks

The canonical laminar cortical architecture that is illustrated in Figures 5, 6, 9 and 10 is specialized within all laminar cortical areas to help carry out the diverse functions that together realize biological intelligence. Such specializations include the 3D LAMINART model for 3D vision and figure-ground perception (Figure 11), the cARTWORD model for conscious speech perception (Figure 13), and the more comprehensive pART architecture that unifies and coordinates many of the brain processes that together realize biological intelligence (Figure 15). These examples elaborate the claim embodied in this article’s title that there exists “a canonical laminar neocortical circuit whose bottom-up, horizontal, and top-down pathways control attention, learning, and prediction.”

The current article has focused upon how feedforward and feedback cortical connections can support the simplest properties of 3D vision; namely, those that depend upon early stages of binocular fusion in cortical layer 3B and boundary grouping in layers 2/3A of V1 and V2. As the 3D LAMINART model illustrates (Figure 11), multiple additional processing stages in cortical areas V1, V2, and V4 are needed to convert the results of binocular fusion in V1 into a 3D surface percept in V4 that supports figure-ground perception.

Interactions with other brain regions are needed to consciously see and recognize this surface percept, so that it can be used as a basis for goal-oriented actions. In particular, *surface-shroud resonances* between V4 and parietal cortex support conscious seeing, and *feature-category resonances* between V4 and inferotemporal cortex support conscious recognition (Grossberg, 2017, 2019).

The current article has also restricted its attention to how feedforward and feedback cortical connections can support vision in response to a stationary scene. It does not incorporate models that clarify how the apparent stability of 3D vision and figure-ground separation are realized when our eyes scan a scene during the learning of view-, position-, and size-invariant recognition categories. How this happens is explained and simulated using the 3D ARTSCAN Search neural model, which has been incrementally developed in a series of articles (McLoughlin and Grossberg, 1998; Cao and Grossberg, 2005, 2012, 2019; Fang and Grossberg, 2009; Fazl et al., 2009; Cao et al., 2011; Foley et al., 2012; Chang et al., 2014; Grossberg et al., 2015).

As noted by Grossberg et al. (2015), achieving active 3D vision and invariant recognition learning requires far more cortical machinery than the visual cortex itself. Indeed, the 3D ARTSCAN Search model “clarifies how perceptual, attentional, and cognitive interactions among multiple brain regions (LGN, V1, V2, V3A, V4, MT, MST, PPC, LIP, ITp, ITa, SC) may accomplish predictive remapping as part of the process whereby view-invariant object categories are learned. These results build upon earlier neural models of 3D vision and figure-ground separation and the learning of invariant object categories as the eyes freely scan a scene. A key process concerns how an object’s surface representation generates a form-fitting distribution of spatial attention, or attentional shroud, in parietal cortex that helps maintain the stability of multiple perceptual and cognitive processes. Predictive eye movement signals maintain the stability of the shroud, as well as of binocularly fused perceptual boundaries and surface representations.” Laminar feedforward and feedback interactions that are variations and specializations of the ones described herein can be used to model all of these processes.

## Author Contributions

The author confirms being the sole contributor of this work and has approved it for publication.

## Conflict of Interest

The author declares that the research was conducted in the absence of any commercial or financial relationships that could be construed as a potential conflict of interest.
